# Neurological Injuries after Calcaneal Osteotomies Are Underdiagnosed

**DOI:** 10.3390/jcm10143139

**Published:** 2021-07-16

**Authors:** David González-Martín, Mario Herrera-Pérez, Jorge Ojeda-Jiménez, Diego Rendón-Díaz, Victor Valderrabano, José Luis Pais-Brito

**Affiliations:** 1 Orthopedic Surgery and Traumatology Service, Hospital Universitario de Canarias, Carretera Ofra S/N, 38320 Tenerife, Spain; davidglezmartin@gmail.com (D.G.-M.); jojedajim@gmail.com (J.O.-J.); diegor07@hotmail.com (D.R.-D.); paisbrito@gmail.com (J.L.P.-B.); 2 Faculty of Health Sciences, Universidad de La Laguna, 38200 San Cristóbal de La Laguna, Spain; 3 Foot and Ankle Unit, Hospital Universitario de Canarias, 38320 Tenerife, Spain; 4 Orthopaedic and Trauma Department, Swiss Ortho Center, Schmerzklinik Basel, Swiss Medical Network, Hirschgässlein 15, 4051 Basel, Switzerland; vvalderrabano@swissmedical.net

**Keywords:** calcaneal osteotomy, nerve injury, approach, complication

## Abstract

The incidence of peripheral neurological injuries related to calcaneal osteotomies reported in the literature is low and often described as occasional. The main objective of this study is to determine the incidence of neurological injuries after calcaneal osteotomies and identify which nerve structures are most affected. This retrospective work included 69 patients. Medical records, surgical protocols, and radiographs were analyzed. All patients were summoned to perform current functional tests (EFAS score and SF-12), and a thorough physical examination was performed systematically and bilaterally. The total incidence of neurological injuries was 43.5% (30/69). The percentage of neurapraxias (transient injuries) was 8.7%, while 34.8% of patients presented neurological sequelae (permanent injuries). The most injured nerve or branch was, in decreasing order: sural nerve, medial plantar branch, lateral plantar branch and medial calcaneal branch. Following the so-called “safe zone” clearly decreases the incidence of sural nerve injury (*p* = 0.035). No significant differences were found between osteotomy site, number of screws, and type of closure and increased neurological injuries. No significant differences were found in the functional tests between the different techniques, nor between patients who presented neurological injuries and those who did not. Neurological injuries after calcaneal osteotomies are underdiagnosed and the incidence is higher than previously reported (43.5%). Such injuries mostly go unnoticed and have no implications in the functional results and patients’ satisfaction.

## 1. Introduction

Calcaneal osteotomy is a procedure performed in routine clinical practice, its main objective is to re-establish the mechanical and load-bearing axis of the foot and ankle, demonstrating good and excellent results in the medium and long term [1,2]. Historically, potential complications associated with this surgery include surgical wound healing problems, infection, discomfort from the osteosynthesis material, over-/under-correction of the deformity, subtalar osteoarthritis, and iatrogenic fractures [1,3,4].

The incidence of peripheral neurological injury after calcaneal osteotomy reported in the literature is low and often described as occasional [5,6]. Lateral nerve structures, such as the sural nerve and lateral calcaneal nerve, are at risk in the lateral approach, while medial structures, such as the posterior tibial nerve and its branches, may be at risk when completing the osteotomy on the medial wall, or when performing the mobile segment displacement to correct the deformity [1,7].

The hypothesis of the present study is that neurological injuries after calcaneal osteotomies are underdiagnosed; therefore, the main objective is to determine the incidence of neurological injuries after calcaneal osteotomies and identify which nerves are most affected.

## 2. Materials and Methods

After approval by the hospital’s ethics board (CHUC_2019_73), a historical cohort study was conducted. Inclusion criteria were patients who underwent calcaneal osteotomy between 2009 and 2019. Exclusion criteria were patients unable to cooperate at interview and/or physical examination, patients < 18 years, and patients with a known pre-existing neuromuscular condition, such as hereditary motor and sensory neuropathy or any sensory deficit. All patients were recruited with a minimum follow-up of 18 months after surgery. All patients signed an informed consent form.

### 2.1. Surgical Technique

All surgical procedures were performed by two senior foot and ankle surgeons (MHO, DRD). The patients were positioned supine on the operating table and received general or spinal anesthesia. The limb was exsanguinated, and the tourniquet inflated to 300 mmHg.

The medial and lateral sliding osteotomy was performed using an oblique incision in the lateral wall of the calcaneus, with a safety margin to respect the sural nerve. After blunt dissection through bone, the dorsal and plantar borders of the calcaneus were identified and protected using two Hohmann retractors. Osteotomy was performed using an oscillating saw; an osteotome can be used to complete the osteotomy on the medial aspect to avoid direct injury to the medial neurovascular structures. A large laminar spreader was used for stepwise mobilization of osteotomy fragments. After appropriate mobilization and relaxation of the surrounding soft tissues, the calcaneal tuberosity was mobilized as planned preoperatively (medial or lateral sliding). A plantar flexed ankle and flexed knee is helpful to relax the entire calf musculature and allow easier displacement. Once the proper position of the calcaneal tuberosity had been achieved, a preliminary fixation was performed from posterior to anterior, avoiding entry into the subtalar joint. After a fluoroscopic check of the final position and location of the K-wires, one or two cannulated, partially threaded, headless screws were used for fixation.

The Zadek osteotomy was performed using the same oblique approach through the lateral wall of the calcaneus. Having identified and marked the dorsal and plantar borders of the calcaneal tuberosity, a dorsal and posterior wedge based oblique osteotomy was performed resecting a bony wedge of 8–10 mm, conserving a plantar hinge. 

The Evans osteotomy or lateral column lengthening osteotomy was performed through an oblique incision just proximal to the calcaneocuboid joint and 1 cm below the tip of the fibula. The muscle belly of the extensor digitorum brevis should be readily visible within the incision and can be reflected from its origin with an L-shaped incision that extends directly onto the calcaneus. Using a blunt retractor, continue dissection and exposure with the release of dorsal and plantar soft tissue from the planned osteotomy site, taking care not to damage the peroneal tendons. The osteotomy is generally 1 to 1.2 cm proximal to the calcaneocuboid joint, which is usually just as the anterior beak begins to flare downward. We performed an incomplete osteotomy perpendicular to the axis of the calcaneus, trying not to break the medial cortex. We then used a laminar spreader into the osteotomy site and determined the size of wedge needed. We finally implanted a titanium wedge plate to produce the lengthening effect.

### 2.2. Postoperative Protocol

A non-weightbearing short leg cast was applied for three weeks, followed by a walker boot for another three weeks, until fusion of the osteotomy was complete.

Medical records, surgical protocols, and pre- and postoperative radiographs were reviewed. All patients were summoned in person to perform current functional tests (EFAS score and SF-12) and to answer some follow-up questions. 

### 2.3. Neurological Examination

A thorough neurological examination was performed systematically and bilaterally before and after the surgical procedure by two experienced foot and ankle surgeons (JOJ, DGM) following the recommendations of the Neurology Department. After the initial examination, another author blinded to the results repeated the neurological examination of the patients to avoid any interpretation bias (JPB).

### 2.4. Exploration of Superficial Sensitivity (Exteroceptive)

**Figure 1:**
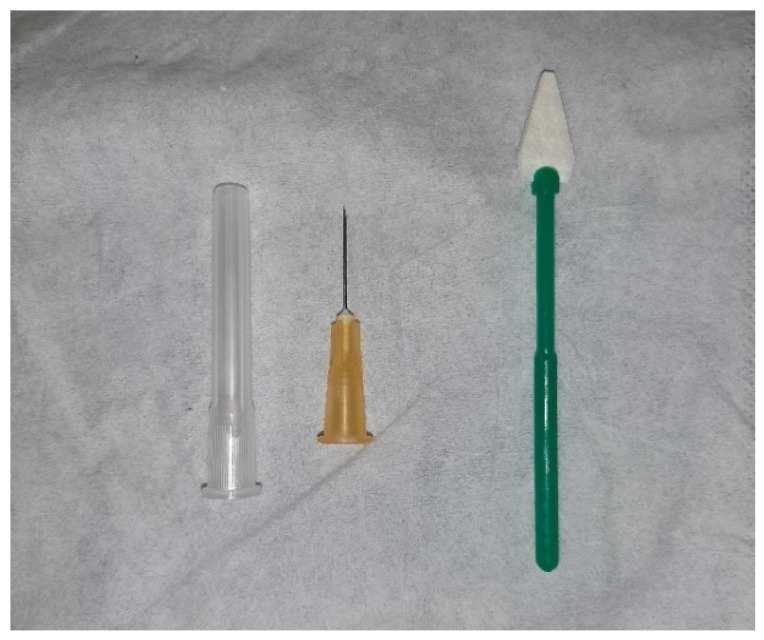
Examination set: intradermal needle and sterile triangular hemostatic suction sponge.

− Touch (Sterile triangular hemostatic suction sponge) (Figure 1): successive touches applied to a comparable range of skin locations in sensitive regions, using simple contact without pressure. The skin was quickly touched two or three times in a row and the subject was asked how many times he/she had been aware of the stimulation. It was considered pathological when the patient was not able to discriminate. Bilateral examination was performed on all patients.
− Algesic (intradermal needle) (Figure 1): touch was applied to different sensitive regions and the patient was asked to discriminate between “touching” or “pricking”. It was considered pathological when the patient was not able to discriminate. Bilateral exploration was performed on all patients.

The exploration was carried out in each of the sensitive regions: posterior tibial nerve, calcaneal branch, medial plantar branch, lateral plantar branch, sural nerve, superficial peroneal nerve, and saphenous nerve.

The type of calcaneal osteotomy (medial or lateral displacement, lateral column lengthening, or Zadek), type of osteosynthesis (cannulated screws, plate, or staples), number of screws, and type of closure (suture, staples, or suture + staples) was analyzed. Surgical wounds were reviewed, and the relative distance proposed by Geng et al. [8] was marked to classify the patient as <1/3 or >1/3 safety distance, depending on whether or not the definition of safety distance, namely an “oblique incision that runs through the point that is no less than one third of the distance from the tip of the lateral malleolus to the posteroinferior margin of the calcaneus”, was respected. After reviewing the preoperative and postoperative radiographs, the osteotomy site was analyzed to assess whether it was in the “safe zone” proposed by Talusan et al. [7]. For example, in a lateral radiograph of the ankle joint, the zone extended 11.2 ± 2.7 mm anterior to the line drawn from the posterior superior apex of the calcaneal tuberosity to the origin of the plantar fascia.

To reduce inter-observer bias, all patients and included radiographs were independently reviewed by at least two of the authors (VV, MHP). All the patients who presented a transient lesion of the sural nerve during the follow-up and were asymptomatic at the time of the present study were considered as neurapraxias; those who presented any permanent injury (touch and/or algesic) were considered to have neurological sequelae. The sum of neurapraxias and neurological sequelae was considered to represent neurological injuries.

Finally, the patients were asked the question: “Knowing the final results of the surgery, if you could go back in time, would you undergo surgery again?”

### 2.5. Statistical Analysis

Proportions were compared by performing chi-square tests, using the Yates correction in 2 × 2 tables. Group comparisons of quantitative and ordinal variables were performed using Student *t*-test, Mann–Whitney or ANOVA, as appropriate. Multiple linear regression or logistic regression was used to model patient functionality if functionality was categorized. Probability values of less than 0.05 were considered significant. Data analyses were performed using the SPSS statistical package (IBM Corp. Released 2017. IBM SPSS Statistics for Windows, Version 25.0, Armonk, NY, USA).

## 3. Results

A total of 76 calcaneal osteotomies in 76 patients were performed between 2010 and 2018, of which seven patients were excluded (one diabetic patient with polyneuropathy, three patients with pre-existing peripheral nerve lesions, one patient below 18 y at the time of the study, and two patients who did not come to outpatient control), thus leaving a total of 69 patients (Table 1). 

Ninety-one percent of patients required an additional procedure at the same time as the calcaneal osteotomy (Broström procedure, ankle arthroscopy, gastrocnemius lengthening, etc.). The diagnoses were: pes cavovarus 60.9%, posterior tibial tendon dysfunction (PTTD) 34.8%, and others (Achilles pathology, post-traumatic deformities, etc.) 4.3% (Table 1). The type of osteotomy can be seen in Table 2.

The incidence of neurological injuries in our series was 43.5% (30/69). The percentage of neurapraxias (transient injuries) was 8.7%, while 34.8% of patients presented neurological sequelae (permanent injuries) (Table 3). Considering all osteotomies (69 patients), the injured nerve or branch in decreasing order was: sural nerve (23 patients), medial plantar branch (21 patients), lateral plantar branch (14 patients), medial calcaneal branch (14 patients), complete posterior tibial nerve (8 patients), superficial peroneal nerve (2 patients).

Six postoperative neurapraxias were diagnosed. The sural nerve was the nerve branch affected in five patients, while one patient suffered a neurapraxia of the posterior tibial nerve trunk. All recovered spontaneously in the first postoperative year. In medial sliding osteotomies, the percentage of neurological injuries was 33.3% (5/15), while in lateral sliding osteotomies the percentage was 52% (22/44). The type of osteotomy performed had a statistically significant influence on the occurrence of neurological injuries (*p* = 0.027).

Failure to respect the safety distance [8] during the approach proved to have a statistically significant effect on the occurrence of a neurological injury of the sural nerve (*p* = 0.035). No statistically significant differences were found between respecting the “safety zone” at the osteotomy site proposed by Talusan et al. or not (*p* = 0.163), the number of screws (*p* = 0.825), or the type of closure (*p* = 0.716) and the occurrence of neurological injuries.

The clinical improvement based on the pre-surgical and current functional tests was significant for the EFAS score (*p* < 0.001), EFAS score sports (*p* < 0.001), and physical SF-12 (PCS-12) (*p* = 0.003). However, it was not significant for the mental SF-12 (MCS-12; *p* = 0.076) (Table 4). Ninety-three percent (64/69) of patients responded that they would undergo surgery again knowing the final result. No significant differences were found in the functional tests between the different techniques, or between patients who presented neurological injuries and those who did not. Finally, presenting a neurological injury was not relevant for the patient when deciding years after surgery whether they would undergo surgery again, knowing the final result (*p* = 0.84).

## 4. Discussion

The most important finding of this work is that we have registered an incidence in neurological injuries higher than previously reported (43.5%). The percentage of neurapraxias (transient injuries) was 8.7%, while 34.8% of patients presented neurological sequelae (permanent injuries). 

Historically, potential complications associated with this surgery include surgical wound healing problems, infections, discomfort from the osteosynthesis material, over-/under-correction of the deformity, subtalar osteoarthritis, and iatrogenic fractures [1,3,4]. As with any type of surgery, injury to the nerves supplying the foot and ankle is always a concern when performing a calcaneal osteotomy [5,6] but, in contrast to our results, the incidence of peripheral neurological injury after calcaneal osteotomy reported in the literature is low and often described as occasional [8,9]. 

Possible causative mechanisms of neurological injuries are direct injury in the lateral approach, injury with the saw (mechanical or thermal) or osteotome in the medial area, traction of the nerve structures (at the time of the displacement of the osteotomy), and reduction in the volume of the tarsal tunnel due to lateral displacement of the medial border, which may compromise its contents [5,6]. The fact that many of these injuries occur in neurological branches that anatomically do not pass through the osteotomy site supports the etiology of indirect mechanism of injury, such as traction or compression [6].

We have registered more neurological injuries with lateral sliding than with medial sliding osteotomies (52% vs 33%), according to the results published by the majority of authors. Van Valkenburg et al. found a neurological injury incidence of 34%, much higher than described previously in the literature [6], and Stødle et al. reported an incidence of 17% [4]. We have identified an injury to the tibial nerve or one of its branches in 21% of cases. Van Valkenburg et al. did not identify differences in the increased risk of neurological injury with the type of osteotomy, the amount of displacement, or prophylactic or non-prophylactic release of the tarsal tunnel [6]. We found differences depending on the type of osteotomy performed, with a higher but not statistically significant incidence of neurological injury after lateral sliding osteotomy compared to medial, although we did not study the amount of displacement or the effect of releasing or not releasing the tarsal tunnel.

Krause et al. reported tibial nerve palsy after lateral displacement osteotomy in two patients treated for Charcot–Marie–Tooth disease. Considering this to be a rare finding, they recommended prophylactic tarsal tunnel release, especially in patients with hereditary peripheral neuropathies [5]. Bruce et al. found that, after lateral sliding osteotomy, the volume of the tarsal tunnel decreased and could potentially damage neurovascular structures travelling through it [3]. Green et al. performed an anatomical study on 22 cadavers, in which they identified the structures at risk during medial displacement osteotomies [9]. These authors described a minimum of two structures that cross the osteotomy site, in most cases branches of the lateral plantar nerve and the posterior tibial artery, and they also recommended caution during completion of the osteotomy at the medial wall to avoid neurovascular injury. For this reason, in our usual technique, the osteotomy is started with a saw but is finished with great care at the medial wall using an osteotome. [9]. 

A higher percentage of lateral plantar nerve injuries is observed at the osteotomy site compared to the medial plantar nerve, as can be seen in our work (20.3% and 15.9%, respectively), although this difference is not statistically significant. 

Minimally invasive techniques have emerged in recent years [10,11,12] with the aim of minimizing incisional complications and reducing pain, although it is essential with this type of technique to ensure minimal damage to local vascular and nerve structures [7,12]. A medial approach has also been described [13] to localize the medial noble structures and thus reduce the incidence of injury, but as with all surgeries it has its advantages and disadvantages and has not displaced the traditional approach.

Talusan et al. conducted a cadaver study looking for the “safety zone” in which calcaneal osteotomy could be performed to help reduce sural, medial plantar and lateral plantar nerve injuries, and was unable to protect the medial or lateral calcaneal branches since there is no consensus on the ideal area for osteotomy [7]. These authors concluded that the safety zone comprises the area formed on a lateral ankle x-ray by a line connecting the posterosuperior apex of the calcaneal tuberosity and the origin of the plantar fascia, and a line 11.2 mm anterior and parallel to it [7]. In contrast to that study, Wills et al. found identical neurological injury rates between osteotomies performed inside and outside the “safety zone” [1]. As with Wills et al., we also found no significant difference between the occurrence of neurological injuries and the performance of the osteotomy inside or outside the “safety zone” (*p* = 0.163). As such, we think that a “safety zone” should really be a zone in which no neurological injuries are found, and as we can see in our sample and in Wills’ publication, this premise is not met. This finding is probably related to a wide anatomical variation of the nerves involved in the procedure [1].

The general principle guiding this technique is that the more anterior the osteotomy is performed, the more the calcaneal tuberosity can be mobilized. However, the sural nerve, the medial plantar branch, and the lateral plantar branch are at high risk if the osteotomy is performed more anteriorly [3,7,14,15]. Kraus et al. reported that lateral translation provides greater correction of varus deformity, although it may cause significant additional shortening of the calcaneus [15]. In addition to possible neurological injuries, the osteotomy should not be too anterior (affecting the subtalar joint and peroneal tendons) or too posterior (the origin of the plantar fascia or the thick fibers of the abductor digiti minimi may inhibit lateral translation of the posterior calcaneal tuberosity) [15]. These authors also recommend using an osteotome to complete the osteotomy on the medial wall (a recommendation that we follow in every case), using the distractor to facilitate removal of the lateral wedge, as this facilitates mobility of the calcaneal tuberosity as well as closure of the osteotomy, and fixing the osteotomy in plantar flexion of the ankle to avoid relaxation of the Achilles tendon [15].

Talusan et al. [7] compared medial sliding osteotomy using the percutaneous versus the open technique. This study showed that the medial and lateral calcaneal branches almost always passed through the osteotomy site and were therefore at risk and had to be protected. Paradoxically, there were fewer injuries to these branches in the percutaneous approach than in the open approach [7]. These authors concluded that, within the proposed safety margin, the sural, medial plantar, and lateral plantar nerves are protected, whereas the medial and lateral calcaneal branches are at risk whatever the location of the osteotomy. However, we found no difference between performing the osteotomy within the proposed “safety zone” and performing it outside of it (*p* = 0.163).

Geng et al. [8] reported that there is no agreement as regards the correct place to perform the lateral approach, although an approach in line with the osteotomy is traditionally used, starting laterally, superior to the calcaneus and extending obliquely distal and inferior to the plantar aspect of the calcaneus [8]. These authors note that the sural nerve is located superficially between the peroneal malleolus and the Achilles tendon and curves forward on the lateral aspect of the hindfoot to continue its pathway to the forefoot. It is therefore vulnerable both during incision and during dissection, osteotomy, and suturing, if not properly approached [8]. They proposed the use of relative rather than absolute measures, as these depend on the size of the patient. Therefore, after dissecting and measuring 20 cadavers, they proposed that the safety distance that should be respected at the level of the skin incision is at least the anterior third of the imaginary line from the tip of the fibula to the most posteroinferior point of the calcaneus at the lateral edge. Our group has recently published a study on this subject, noting that respecting the “safety distance” reduces the occurrence of sural nerve injuries in a statistically significant manner (*p* = 0.035) [16].

Prophylactic tarsal tunnel release should logically protect against nerve injury in lateral sliding osteotomies, as several studies have established that the direction and amount of displacement has a direct impact on tarsal tunnel volume [3,13]. Nevertheless, Van Valkenburg et al. reported that not only was there no decrease in neurological injury in patients who underwent tarsal tunnel release, but that there was an increase in the incidence of neurological injury [6]. However, these authors noted that this may have been due to bias, as they only found a significant difference in the occurrence of neurological injuries in those patients who underwent tarsal tunnel release after osteotomy, and it is likely that release after osteotomy was performed in those patients where there was a higher risk of injury, i.e., with greater deformity, or greater lateral displacement, which in itself predisposed them to a higher risk of injury [6]. 

Bruce et al. measured tarsal tunnel volume by MRI after both medial and lateral sliding osteotomy [3]. They proved that tarsal tunnel volume decreased in lateral displacement osteotomies by 14–21%, depending on the amount of displacement. In our study, the type of osteotomy performed had a statistically significant influence on the occurrence of neurological injuries (*p* = 0.027), although no statistically significant differences were found between medial and lateral sliding osteotomies and the occurrence of injuries to the tibial nerve or its branches. Another study that used computed tomography to measure tarsal sinus volume [4] reported a decrease in volume, although this was not related to an increase in neurological injuries. The authors of that study showed that one patient out of a total of 15 suffered transient neurapraxia of the sural nerve, which resolved spontaneously in less than six months, and three patients (20%) presented sural nerve sequelae [4]. In the present study, five patients (7%) presented sural nerve neurapraxia, while 18 patients (26.1%) presented permanent sequelae of the sural nerve. We did not measure the volume of the tarsal tunnel, nor did we measure the amount of medial or lateral displacement.

In light of the above, the position on the preventive release of the tarsal tunnel is currently not clear as some studies are in favor [3] and others are against [6,13] performing this per protocol. However, there is greater consensus, regarding releasing the tarsal tunnel prophylactically in patients with severe varus deformities, such as Charcot-Marie-Tooth, to avoid potential complications, and this is the case in our center, although the level of evidence is not high [4,5].

In the methodology of our study, we wanted to include a minimum follow-up time for patients, to confirm that we were really talking about neurological sequelae and not transient neurapraxia, i.e., a self-limiting nerve compression or contusion, over time. To define the minimum time, we based our decision on published studies of neurological recovery after foot and ankle surgery, in which the vast majority of neurapraxias recovered in the first six months and no differences were found in the examination after the ninth month [17]. Wills et al. also established a minimum six-month follow-up [1]. We thought it appropriate to increase the margin of recovery, therefore the minimum follow-up time was 18 months, thus making this the only study in the literature concerning neurological injuries in the medium–long term. 

In their work on the incidence of superficial peroneal nerve injury after ankle fractures, Redfern et al. [18] reported that many of the patients who were diagnosed with neurological injury had not been previously diagnosed until the examination for the study, as is the case in our work. The patient often accepts a sensory alteration as normal after surgery and does not complain to their doctor. Moreover, this often does not cause a disability in the patient, as can be seen from the clinical improvement based on the functional tests (EFAS score, EFAS sports, and physical SF-12), which show no significant differences between the different techniques, or between patients who present neurological injuries and those who do not. Therefore, when explaining the risks of surgery to a patient, we should point out that, despite being a careful and regulated technique [19], neurological injuries are a risk to be taken into account, with the risk of dysesthesia or numbness in the injured sensory area.

Finally, many calcaneal osteotomies require concomitant procedures [13,14], as can be seen in our series, where 91% of patients underwent some ancillary procedure (Broström, ankle arthroscopy, gastrocnemius lengthening, etc.) in the same surgical episode. It is possible that the concomitant procedures could eventually cause some neurological injury, however, in our work, we wanted to study specifically all the neurological complications directly related to the calcaneal osteotomy area and the main nerves around it.

There are several weaknesses and strengths of the current study that need to be pointed out. First, the cohort size was relatively small, although it represents the largest series published so far. Second, in no case was a neurophysiological test used to confirm the degree and severity of the nerve injury, which would have been desirable, since the authors consider that the detailed clinical neurological examination, as explained in the Methodology section, provides us with more information than the neurophysiological test itself. Furthermore, in order to reduce inter-observer bias, all patients included in the study were examined at the outpatient clinic by at least two of the authors. Third, when collecting data from the clinical history, it is possible that some data were not collected in the history and were therefore not included in the study, thus it is possible that some neurapraxia was overlooked. Fourth, this is the first clinical study in which all possible neurological injuries after calcaneal osteotomy are studied, since previous authors have analyzed only medial or lateral injuries, thus allowing us to determine the real global incidence of neurological injuries in this type of surgery. However, we are unable to explain the cause of the injury, as it could be the result of an ischemic injury from the torniquet, positioning in surgery, or possibly the boot/cast during the post-surgery period. Finally, most of the studies on neurological injuries have been carried out on cadavers, and only a few, such as the present work, have been carried out in vivo.

An important finding of the current study is that the incidence of neurological injuries after calcaneal osteotomies is higher than previously reported. This fact is important and should be explained to patients prior to surgery and included in the informed consent. Finally, even though this injury mostly goes unnoticed and has no implications in the final functional results, there is a minority of patients in whom it does cause significant discomfort in their daily activities.

## 5. Conclusions

Neurological injuries after calcaneal osteotomies are underdiagnosed and the incidence is higher than previously reported (43.5%). The percentage of neurapraxias (transient injuries) was 8.7%, while 34.8% of patients presented neurological sequelae (permanent injuries). The most frequently injured nerve or branch was, in decreasing order: sural nerve, medial plantar branch, lateral plantar branch and medial calcaneal branch. Such injuries mostly go unnoticed and have no implications in the functional results and patients’ satisfaction.

## Figures and Tables

**Table 1:** Case demographics and distribution.

Variables	Patients (*n* = 69)
Age	48.81 years (R, 13–79 years)
Sex	Female: 65.2%; Male: 34.8%
Laterality	Left foot 50.7%; Right foot 49.3%
Diagnosis	Pes cavovarus: 60.9% Posterior tibial tendon dysfunction (PTTD): 34.8% Others: 4.3%
Mean follow-up time	47 months (R, 18–114 months)

**Table 2:** Type of osteotomy.

Type of Osteotomy	Patients (%) (*n*/N)
Medial sliding osteotomy	21.7 (15/69)
Lateral sliding osteotomy	60.9 (42/69)
Lateral column lengthening (Evans)	14.5 (10/69)
Zadek’s osteotomy	2.9 (2/69)

**Table 3:** Neurological sequelae (permanent injuries).

Neurological Sequelae					
	Medial Sliding	Lateral Sliding	Evans	Zadek	Total
*Complete posterior tibial nerve*	2/15 (13.3%)	3/42 (7.1%)	1/10 (10%)	1/2 (50%)	7/69 (10.1%)
*Calcaneal branch*	2/15 (13.3%)	9/42 (21.4%)	1/10 (10%)	1/2 (50%)	13/69 (18.8%)
*Medial plantar branch*	2/15 (13.3%)	7/42 (16.7%)	1/10 (10%)	1/2 (50%)	11/69 (15.9%)
*Lateral plantar branch*	3/15 (20%)	9/42 (21.4%)	1/10 (10%)	1/2 (50%)	14/69 (20.3%)
*Sural nerve*	3/15 (20%)	12/42 (28.6%)	1/10 (10%)	2/2 (100%)	18/69 (26.1%)
*Superficial peroneal nerve*	0/15 (0%)	1/42 (2.4%)	1/10 (10%)	0/2 (0%)	2/69 (2.9%)
*Saphenous nerve*	0/15 (0%)	0/42 (0%)	0/10 (0%)	0/2 (0%)	0/69 (0%)

**Table 4:** Presurgical vs Current functional test (Wilcoxon test).

Functional Test		
Pre-surgical EFAS score (0–24)	10.04 (sd 5.650)	*p* < 0.001
Current EFAS score (0–24)	17.06 (sd 5.882)	
Pre-surgical EFAS sport score (0–40)	14.94 (sd 10.136)	*p* < 0.001
Current EFAS sport score (0–40)	24.52 (sd 10.885)	
Pre-surgical SF-12 (PCS-12)	34.38 (sd 7.33)	*p* = 0.003
Current SF-12 (PCS-12)	38.30 (sd 5.49)	
Pre-surgical SF-12 (MCS-12)	43.09 (sd 6.98)	*p* = 0.076
Current SF-12 (MCS-12)	45.15 (sd 6.09)	
